# Insulin resistance and adverse metabolic profile in overweight/obese and normal weight of young women with polycystic ovary syndrome

**DOI:** 10.22088/cjim.9.3.260

**Published:** 2018

**Authors:** Maryam Gholinezhad, Masoumeh Gholsorkhtabaramiri, Sedigheh Esmaeilzadeh, Azita Ghanbarpour

**Affiliations:** 1 1. Infertility and Reproductive Health Research Center, Health Research Institute, Babol University of Medical Sciences, Babol, Iran; 2 Clinical Research Development unit of Ayatollah Rouhani Hospital, Babol University of Medical Sciences, Babol, Iran

**Keywords:** Insulin resistance, Obesity, Polycystic ovary syndrome, Overweight

## Abstract

**Background:**

Polycystic ovary syndrome (PCOs) is an endocrine-metabolic disorder. This study intends to determine the comparison of insulin resistance (IR) and metabolic disturbance in overweight/obese and normal-weight of young women with polycystic ovary syndrome.

**Methods:**

Using a comparative cross-sectional study design in 2015, 27 normal weight (18<BMI<25) and 85 overweight/obese (BMI≥25) aged 18 and 35 underwent clinical measures of HOMA (IR) as insulin resistance and QUICKI as insulin sensitivity tools in Fatemezahra Infertility Research Center of Babol. Lipid profile and hormonal parameters were evaluated between two groups.

**Results:**

112 women with PCOS participated in this study. The mean age was 22.4±3.48 years in the normal PCOS group (n=27) and 24.4±5.06 years in the overweight/obese PCOS patients (n=85). BMI had a significant straight correlation with insulin resistance (p<0.001) and a negative correlation with insulin sensitivity (p<0.001). BMI showed a straight stronger correlation with triglyceride (TG) (p<0.001) and LDL cholesterol (<0.05) and a stronger reverse relationship with SHBG (p<0.001). In overweight/obese group, 91.7% (48) of the women showed insulin resistance (HOMA>3.15) vs. 8.3% (5) in the normal group (P<0.001). 82.4% (62) of the overweight/obese group revealed low insulin sensitivity (QUICKI<0.34) while this value was 17.6 % (13) within their lean counterparts (p<0.001). In the study group, 89.7 % (54) showed elevated fasting insulin concentration (>13µU/ml) vs. 10.3% (7) in the control group (p<0.001).

**Conclusions:**

Overweight/obese PCOs patients revealed higher insulin resistance and lower insulin sensitivity, and also greater TG and LDL cholesterol. Priority of management of insulin resistance and lipid profile should be considered on identifying these potentially major complications.

Insulin resistance (IR) is a growing public health concern worldwide. Co-occurrence of IR, as a well-established contributor of metabolic syndrome is regarded in women with PCOs (1). Glucose intolerance in PCOS women occur at an earlier age than in the normal ovary women (in the 3rd-4th decades of life) (2). The PCOs women may involve several fold more likely to have increased risk of aggravating type II diabetes and an excess risk of impaired glucose tolerance, and more suffering from diabetes at young age (3-8). Approximately, half of the women with PCOs are overweight or obese. They involve 10-year incidence of major weight gain (9). Obesity aggravates extended medical implications in women with PCOs. On the other hand, greater prevalence of metabolic abnormalities is seen in the presence of obesity alongside PCOs phenotype (10-12).

In a study, 57% of overweight/obese women with PCOs are insulin resistant, while the prevalence was 9.3 % in the lean counterparts. In addition, insulin sensitivity (IS) was reduced 50% in obese PCOs from that in lean controls (13). Even augmenting BMI by 1 kg/m^2^ reduces IS indexed further in PCOs patients versus in controls (14). Recent evidence has suggested that adiposity contributes as an additional component to IR in obese PCOs (15-18), however, not a defining criteria for PCOs (14). Hence, weight loss through lifestyle modification improves IR and reduces and detracts PCOS clinical manifestation intensity (19).

Although, IR may be a multifactorial feature; particularly race and age have an important role as well as BMI (20). Higher fasting insulin and lower IS were seen in South Asians, African Americans and Hispanic women with PCOs at a younger age with severe manifestations than Caucasians (21-22). IR is an independent risk factor for clinical, metabolic, reproductive, and psychological problems of overweight/obese PCOs women (23) from diabetes mellitus, hypertension, cardiovascular disease to resistance to weight loss, ovulation disturbance, irregular menstruation, hyperandrogenism, infertility, increased miscarriage rate and even psychological distress (24), yet a considerable amount of respective literature has been published on IS regarding Caucasian women. Thus, little reliable evidence in the Middle East with its related races or the measurement tools for calculating IR was not attributable. 

In this study, we assess the comparison of insulin resistance and some biochemical features in lean and overweight/ obese young women with PCOs. 

## Methods

**Participants:** The Ethics Committee of Babol University of Medical Sciences approved the study protocol. This comparative cross-sectional study was conducted on a total of 112 PCOs (27 women with normal weight and 85 overweight obese women) aged 18 to 35, who referred to the PCOs clinic of Infertility Research Center in Babol (Iran), from April to September 2015. 

The sample size was calculated according to previous related papers. We recruited the samples amongst the women with PCOs admitted in the clinic. All participants were or their first visit prior to the initiation of any infertility co-treatment or main treatment. PCOs was defined according to the 2003 Rotterdam consensus criterion (25). Diagnosis of PCOs was based on ultrasound, clinical and biochemical criteria. Ultrasound criteria were used to detect polycystic ovaries, defined as the presence of at least 12 follicles of 2 to 9-mm diameter, and/or increased ovarian volume (> 10 ml). Clinical criteria included amenorrhea or oligomenorrhea (a cycle length >35 days or six menstrual periods per year). Moreover, patients who were diagnosed as PCOs had hirsutism assessed using Ferriman and Gallway score system (≥ 8), acne and alopecia. Biochemical criteria included elevated menstrual LH/FSH ratio (>2) or elevated serum testosterone (>2.8) nmol/L and or biochemical signs of hyperandrogenism including: increased circulating level of total or free testosterone, or dehydroepiandrosterone sulfate (DHEAS), while the other causes of hyperandrogenism had been excluded. 

**The patients with the following conditions were also excluded from this study:** pregnancy, hyperprolactinemia, thyroid dysfunction, hypertension, gastrectomy, cigarette smoking, chronic alcohol consumption, contraceptive pills and anti-obesity, strenuous physical activity and the women who were not exposed to any infertility treatments. The selected PCOs patients were divided into two groups based on BMI; 18-25 normal weight, ≥25 overweight and obese. BMI was calculated using the formula: [weight/height^2^; (kg/m^2^)]. Waist circumference to hip circumference ratio was calculated in all patients. The patients were matched on the basis of age.

**Laboratory assays:** We preferred to choose HOMA (IR) (homeostatic assessment of IR tool) and fasting insulin in the assessment of IR and QUICKI (quantitative IS check index to investigate IS for their simplicity and identical diagnostic accuracy in our participants at risk of diabetes (26-28). HOMA-IR and QUICKI were calculated according to the following formula: HOMA-IR= [fasting insulin (µIU/ml) × fasting glucose (mg/dl)]/405 and QUICKI = [(1/(log fasting insulin (µIU/ml) +‏ log fasting glucose (mg/dl)] indexes (29). IR specific for PCOs women was defined as HOMA-IR greater than 3.15 (13), abnormal fasting insulin (<7 and >13 mIU/l) and fasting plasma glucose >100.9 µg/dl (5.7 mmol/L or 5.6 mmol/L or more) (30). All hormonal and biochemical experiments were performed in a private pathobiology laboratory in Babol, Iran. Blood samples (~ 10 mL) were collected from each patient after an overnight fast for at least 12 hr. The samples were immediately cooled, serum and plasma were separated within 1 h and stored at -20° C until assayed. Biochemistry analysis including FBS; fasting blood sugar (mg/dl), fasting insulin (µg/dl) cholesterol (mg/dl), triglycerides (mg/dl), HDL-C; high-density lipoprotein-cholesterol (mg/dl), LDL-C; low-density lipoprotein- cholesterol (mg/dl) were measured with Azmoon kit ( Pars Azmoon kit, Pars Azmoon Inc., Tehran, Iran) by the spectrophotometry method (Hitachi 911 Chemistry analyzer, Germany). We used optimal cutoff TG≥68.5 for normal weight and ≥100.5 mg/dl for overweight/ obese women as a marker for predicting IR according to Park (31). Serum insulin (µU/ml) was analyzed via IRMA: immunoradiometric assay kit (BI-insulin IRMA; Bio-Rad, Marnes la Coquette, France). 

Hormonal analysis including serum 17-OH-progestrone level (ng/ml) was measured using a radioimmunoassay (RIA‎). Serum levels of prolactin (mlu/L), testosterone (ng/ml), DHEAS; dehydroepiandrosterone sulphate (µg/dl), FSH; follicle-stimulating hormone (mIu/ml), LH; luteinizing hormone (mIu/ml) were performed with chemiluminescence immunoassay kit (CLIA). TSH; thyroid stimulating hormone (mIu/mL) was determined with IRMA: immunoradiometric assay. SHBG; sex hormone binding globulin (nmol/L) was measured by electrochemiluminescence assay kit (ECL). Free testosterone (pg/mL) was analyzed using ELISA Kit.

Statistical analysis: Statistical tests were performed using SPSS 16 for Windows software (SPSS Inc., Version 18, Chicago, IL, USA). The difference mean of all parameters between the study groups were done using t- test. Log transformation analysis of correlation between BMI and hormonal and biochemical tests was performed using Pearson’s bivariate correlation coefficient. The results were expressed as mean±S.D. For testing the differences in the frequencies of classified HOMA (IR), QUICKI, and fasting insulin in normal and overweight/obese PCOs women, chi-square was used. For all analyses, a 2-tailed p-value of 0.05 or less was considered statistically significant. Data were presented as means and standard deviations.

## Results

112 women with PCOs participated in this study. The study group included 85 (75.9%) overweight /obese PCOs subfertile women with BMI ≥25 and control group 27(26.5%) with normal weight PCOs with BMI<25. The anthropometric parameters of age, BMI, waist/hip ratio, hirsutism by Ferriman-Gallwey score of normal, overweight/ obese PCOS participants were summarized in table 1. 

**Table 1:** Anthropometric parameters in normal and overweight/obese PCOs women

	**Normal weight** **(n=27)**	**Overweight-obese** **(n=85)**
	**Mean±SD**	**Mean±SD**
Age (years)	22.4±3.48	24.4±5.06
BMI (kg/m^2)^	22.37±2.26	30.86±3.84
Waist/hip ratio	0.76±0.09	0.86±0.94
Hisrsutism^1^	12.11±6.36	12.85±5.49

Data was shown by descriptive test.

1 Ferriman and Gallway score

Mean age was 22.4±3.48 years in the normal PCOs group (n=27) and 24.4±5.06 years in the overweight/obese PCOs patients (n=85). Correlation between variables: Correlation analysis between BMI and biochemical, hormonal parameters of women with PCOs is presented in table 2. Table shows the variables which are in correlation with BMI. It is apparent that the strongest straight correlation is between BMI with fasting insulin r=0.55. 

Additionally, it is quite revealing that BMI had a significant positive correlation with HOMA (IR), r= 0.47, and also a significant reverse correlation with QUICKI, r=-0.53 (table 2). 

**Table 2:** Correlation analysis between BMI and hormonal and biochemical parameters of women with PCOs

**Dependent variable**	**R **	**P value** *
LH (mIU/ml)	-0.12	0.04
SHBG (nmol/L)	-0.48	<.001
Triglyceride (mg/dl)	0.39	<.001
LDL-cholesterol (mg/dl)	0.24	0.01
Fasting insulin (µU/dl)	0.55	<.001
QUICKI	-0.53	<.001
HOMA(IR)	0.47	<.001

HOMA-IR: Insulin resistance calculated using HOMA model

QUICKI: Insulin sensitivity calculated using QUICKI model

* The data were analyzed by logistic regression.

Metabolic assessment and hormonal profiling of two groups of PCOs patients divided based on BMI was illustrated in table 3. Overweight- obese PCOs women significantly achieved higher HOMA-IR and QUICKI (p<0.001), (p<0.001), respectively (table3). The difference in lipid profile containing triglyceride and LDL cholesterol values were higher in the overweight/obese PCOs women reaching the level of statistical significance (p<0.05), (p<0.05), respectively. Also, HDL was lower in the overweight/obese women than the lean PCOs group (p≤0.05) (table 3). 

**Table 3:** Biochemical parameters in normal and overweight/obese PCOS women

	**Normal** **(n=27)**	**Overweight-obese** **(n=85)**	**P-value** *
	**Mean±SD**	**Mean±SD**	
FSH (mIU/ml)	6.72 ± 2.03	6.52 ± 1.72	0.62
LH (mIU/ml)	8.69 ± 3.96	8.3 ± 4.28	0.65
LH/FSH ratio	1.29 ± 0.49	1.29 ± 0.8	0.99
Free-testosterone (pg/ml)	2.85 ± 1.47	2.79 ± 1.06	0.78
Testosterone (ng/ml)	1.08 ± 1.67	0.9 ± 1.13	0.54
17-OH-progesterone (ng/ml)	1.31 ± 1.79	1.14 ± 0.91	0.587
DHEAS (µg/dl))	179.09 ± 86.36	172.7 ± 73.64	0.71
SHBG (nmol/L)	45.24 ± 29.6	29.19 ± 13.31	0.01
TSH (mIU/ml)	2.18 ± 1.24	2.3 ± 1.17	0.67
Prolactin (mlU/L)	374.74± 185.8	265.11± 152.79	0.02
Fasting insulin (µU/ml)	10.87 ± 6.99	16.78 ± 7.41	<0.001
Fasting glucose (mg/dl)	87.87 ± 9.27	89.01 ± 11.04	0.6
Triglyceride (mg/dl)	98.37 ± 27.46	139.4 ± 64.15	0.03
Total cholesterol (mg/dl)	175.77 ± 39	184.08 ± 37.49	0.33
LDL-cholesterol (mg/dl)	91.52 ± 8.92	104.52± 23.71	0.02
HDL-cholesterol (mg/dl)	58.27 ± 8.78	53.71± 12.84	0.01
QUICKI < 0.34	0.37±0.03	0.31±0.01	<0.001
HOMA (IR) ≥ 3.15	2.01± 0.38	5.15 ± 1.93	<0.001

Overweight (body mass index [BMI] ≥25 kg/m^2^

HOMA-IR: Insulin resistance calculated using HOMA model

QUICKI: Insulin sensitivity calculated using QUICKI model

* t-Test.

After applying TG cutoff (≥68.5 for normal weight and ≥100.5 mg/dl for overweight/ obese) that was defined by Park as a marker for predicting IR (HOMA-IR≥2.5) (31), the results revealed that 18.2% (2) women in the normal group had TG greater than 67.5, while TG≥100.5 mg/dl was 75.3% (73) amongst the overweight/obese women. The difference was not significant. Moreover, hormonal results indicate that overweight/obese PCOS patients had lower SHBG and elevated prolactin concentration (p< 0.05, p<0.001). Glucose findings: Table 4 demonstrated that the prevalence of IR (HOMA >3.15) revealed 47.1 %( 53) and HOMA-IR≤ 3.15 was 49% (57) irrespective of whether they were normal, overweight/obese that displays significant difference (P<0.00) (table 4). 

**Figure 1:**
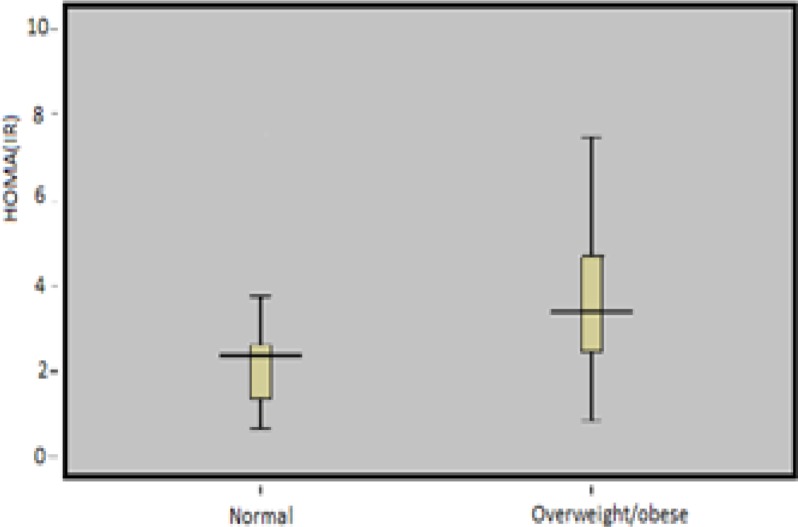
Mean Homa(IR) data for normal control, overweight/obese PCOs women. Groups were significantly different from each other (p<0.001), (p<0.001), respectively.

Fig 1 revealed higher mean of Homa (IR) data for overweight/obese vs. normal control PCOS women 72.5% (76) of our participants had quantitative IS check index (QUICKI)<0.34 and 27.5%(32) had QUICKI ≥0.34 without considering BMI classification. More than 80% of overweight/obese PCOS patients showed low IS (QUICKI <0.34) and over 90% of these groups of patients revealed high IR (HOMA>3.15) (table 2). 

**Figure 2:**
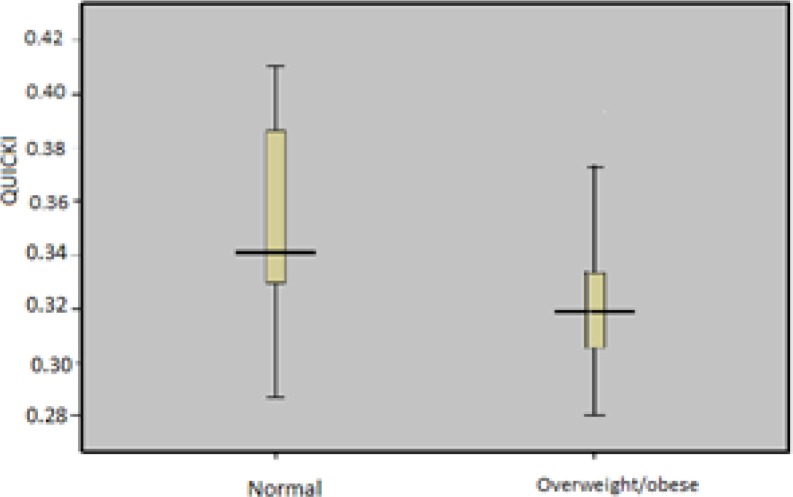
Comparison of QUICKI in BMI- classified (normal and overweight/obese) PCOs women. Groups were significantly different from each other (p<0.001), (p<0.001), respectively

Additionally, the elevated mean of QUICKI in overweight/obese vs. normal weight PCOS women was displayed in fig 2. To assess fasting blood sugar (FBS), we applied optimal cutoff >100.9 mg/dl (5.7 mmol/L or 5.6 mmol/L or more) for fasting plasma glucose according to Gagnon (32). FBS in 89.2% (95) was less than 100.9 mg/dl and 10.8% (14) was over apart from the BMI classification (table 4). 

Comparison of the diagnosis with high accuracy of two cutoff fasting insulin values (<7 and ≥13 µg/dl) is represented by Lunger (30). In our study, 15.7% (18) of women had fasting insulin less than 7 µg/dl and 56.9 % (61) of women ≥13 µg/dl irrespective of BMI classification. Over 85% (54) of our overweight/obese PCOS patients revealed fasting insulin≥13 versus 10.3 % (7) in the normal PCOs control. 

Nonetheless, the women with insulin level<7 µg/dl are similar within two groups.

**Table 4:** Insulin parameters in normal and overweight/obese PCOs women

		**Normal**	**Overweight/Obese**	**Pvalue**
Fasting glucose (mg/dl)	<100.9	27.5(26)	72.5(68)	0.5
	≥100.9	18.2(4)	81.8(12)	
Fasting Insulin	<7	50%(8)	50%(8)	<0.001
(µU/ml)	≥ 7 and <13	46.4%(14)	53.6%(17)	
	≥13	10.3%(7)	89.7%(54)	
HOMA(IR)	≤3.15	38%(22)	62%(35)	<0.001
	>3.15	8.3%(5)	91.7%(48)	
QUICKI	≥0.34	50%(16)	50%(16)	<0.001
	<0.34	17.6%(13)	82.4%(62)	

The values is significant at P≤0.05, rates were compared with chi- square test.

Overweight and obese (body mass index [BMI] ≥25 kg/m^2^

PCOs; Polycystic ovarian syndrome

HOMA-IR: Insulin resistance calculated using HOMA model

QUICKI: Insulin sensitivity calculated using QUICKI model

## Discussion

In the current study, we found that over 90% of the overweight/obese PCOs patients showed high IR and over 80% of them showed decreased IS. Moreover, 85% of the overweight/obese PCOs participants demonstrated elevated fasting insulin concentration.

Morciano’s classified 72% of Italian overweight/ obese women as insulin resistant compared with 26.3% of the lean counterpart and the difference was significant (27). Morciano’ findings are in agreement with those obtained by us; however, the discrepancy in the rates could be attributed to the method of IR calculation and ethnic variation. He evaluated IR through hyperinsulinemic- euglycemic clamp. Besides, our findings are in accordance with the study by Stepo et al. in Australia indicating that 95% of overweight/ obese PCOs and 75% of lean PCOS women are in the risk of IR (17). He also evaluated IR through hyperinsulinemic- euglycemic clamp. Yet, Stepo’ finding need doubtless be scrutinized whereas he did not mention his samples were taken from similar races or not. Wijeyaratne et al. found IS to be lower in women of the Middle East (22). Taking into account racial homogeneity, we applied IR specific cutoff value ≥ 3.15 for PCOs women in which Alebic et al. recommended in their paper (13). Behboudi-Gandevani et al. in an earlier review article revealed that the mean of HOMA IR in Iranian obese PCOs group was 4.38 which are more than their non- obese counterparts and the difference was significant. They found no evidence of any effects of obesity on IR sensitivity (33). Another study in Iran demonstrated that 36% of overweight/ obese PCOs patients had insulin resistance. Their excess rate may be somewhat limited by excess HOMA (IR) (≥3.8) that they considered as insulin-resistant criteria (34).

Irrespective of age and body mass index, race and lifestyle (diet) may have a pivotal role in the overall prevalence of IR. Glintborg et al. in their study demonstrated that the risk of diabetes was comparable between the Middle East and Caucasian populations in Denmark, while the frequency of fasting insulin was enhanced in the Middle East PCOS women (35). With respect to the adjacent countries which may have similar races to Iran, Jamil et al. reported IR was 42.6% (cutoff value of HOMA-IR ≥3.8 for PCOS women) within Iraq’s PCOs women and Tabassum et al. in Pakistan that his obtained prevalence of IR was 34.80% (36-37). The data in the first study approximately accorded with what we found in our study. Possible explanations for the difference in the recent mentioned study by Tabassum can be attributed to the type of applied measurement tools of IR that was calculated by means of insulin ratio.

Another important finding was that 85% of overweight/ obese PCOs patients had elevated fasting insulin concentration with highly statistically significance compared with normal PCOs controls. Schachter addressed IR identified in 53.5% (83/155) of PCOs patients (38). He calculated fasting insulin >19.1 mIU/l. This low result is likely to be related to the rather range of fasting insulin level that they considered as criterion of insulin level (29).

Contrary to expectations, this study did not find a significant difference between fasting glucose concentration in normal weight as opposed to overweight/obese; however, this value was enhanced in the women with adiposity. This result further supports the idea of Gagnon that claimed screening for abnormal glucose tolerance when the results of a fasting plasma glucose test are 5.6 mmol/L or more are not reasonable for women with polycystic ovary syndrome (32). Also, Palmert et al.’s study seems to be consistent with our study and identified that fasting glucose level is a suboptimal criterion at least for impaired diabetes diagnosis (39). On the question of other metabolic profile, this study detects the evidence that women with greater BMI have less SHBG. These findings support previous research into this brain area that hyperinsulinemia exacerbate androgen production as well as declining sex hormone-binding globulin (40). We also observed excess triglyceride and LDL cholesterol in overweight/obese women versus normal weight ones. This result is in consistence with Layegh and et al. (41). Park et al. in their study investigated that TG≥100.5 in obese group is a best marker for predicting IR (HOMA-IR≥2.5) in Korean obese and non-obese PCOs women. In our study, more than 75% of the overweight/obese women had TG≥100.5 and predicted IR (HOMA-IR>3.15). The result needs to be considered cautiously for the greater HOMA-IR that we applied for calculation IR according to Park (31). A limitation consist of not using glucose tolerance test (GTT) and comparing that with other respective tests to entirely characterize the IR owing to having not enough budget. Further research is required to determine the racial variability in intrinsic IR and BMI-related IR.

In conclusion majority of the overweight/obese PCOS patients showed higher insulin resistance, lower insulin sensitivity and elevated fasting insulin concentration vs. their normal weight counterparts. PCOs women are more prone to adiposity and excess body mass index was associated with IR. Consequently, IR disorders androgen profile and threatens metabolic and reproductive health across life span, accordingly, health strategy needs to be screened for the diagnosis of IR in all young PCOs overweight/obese women. 
